# Application of WHO’s guideline for the selection of sentinel sites for hospital-based influenza surveillance in Indonesia

**DOI:** 10.1186/1472-6963-14-424

**Published:** 2014-09-23

**Authors:** Ni Ketut Susilarini, Martahan Sitorus, Catharina Yekti Praptaningsih, Ondri Dwi Sampurno, Arie Bratasena, Ester Mulyadi, Roselinda Rusli, Ahmad Fandil, Amalya Mangiri, Hana Apsari, Edy Hariyanto, Gina Samaan

**Affiliations:** National Institute of Health Research and Development, Ministry of Health, Jakarta, Indonesia; Disease Control and Environmental Health, Ministry of Health, Jakarta, Indonesia; Influenza Division, United States Centers for Disease Control and Prevention, Jakarta, Indonesia

**Keywords:** Surveillance, Indonesia, Pneumonia, System, Respiratory disease, Severe acute respiratory infection, Influenza-like illness

## Abstract

**Background:**

A sentinel hospital-based severe acute respiratory infection (SARI) surveillance system was established in Indonesia in 2013. Deciding on the number, geographic location and hospitals to be selected as sentinel sites was a challenge. Based on the recently published WHO guideline for influenza surveillance (2012), this study presents the process for hospital sentinel site selection.

**Methods:**

From the 2,165 hospitals in Indonesia, the first step was to shortlist to hospitals that had previously participated in respiratory disease surveillance systems and had acceptable surveillance performance history. The second step involved categorizing the shortlist according to five regions in Indonesia to maximize geographic representativeness. A checklist was developed based on the WHO recommended attributes for sentinel site selection including stability, feasibility, representativeness and the availability of data to enable disease burden estimation. Eight hospitals, a maximum of two per geographic region, were visited for checklist administration. Checklist findings from the eight hospitals were analyzed and sentinel sites selected in the third step.

**Results:**

Six hospitals could be selected based on resources available to ensure system stability over a three-year period. For feasibility, all eight hospitals visited had mechanisms for specimen shipment and the capacity to report surveillance data, but two had limited motivation for system participation. For representativeness, the eight hospitals were geographically dispersed around Indonesia, and all could capture cases in all age and socio-economic groups. All eight hospitals had prerequisite population data to enable disease burden estimation. The two hospitals with low motivation were excluded and the remaining six were selected as sentinel sites.

**Conclusions:**

The multi-step process enabled sentinel site selection based on the WHO recommended attributes that emphasize right-sizing the surveillance system to ensure its stability and maximizing its geographic representativeness. This experience may guide other countries interested in adopting WHO’s influenza surveillance standards for sentinel site selection.

## Background

Influenza surveillance has been instrumental in advancing global understanding of the disease, monitoring changes in antigenicity of different subtypes and guiding influenza vaccine strain selection [1, 2]. With the emergence and circulation of the avian influenza A/H5N1 virus in several countries, including in Indonesia, influenza surveillance became a global public health priority to prepare for an ensuing influenza pandemic. During the influenza A/H1N1pdm09 pandemic in 2009, influenza surveillance systems enabled monitoring of pandemic disease trends, severity and mortality [3–5].

In July 2012, the World Health Organization (WHO) released updated standards for influenza surveillance [6]. This guideline built on the lessons learnt from the 2009 influenza pandemic and outlined both the epidemiological and virological surveillance objectives, as well as revised case definitions and minimum data set requirements. The primary objective of sentinel influenza surveillance systems as stated in the guideline is to monitor influenza activity rather than to detect outbreaks. Sentinel influenza surveillance systems can provide valuable information about epidemiological trends including seasonality, age groups affected and groups at risk of severe disease. Sentinel systems can also provide information about virological trends, and to identify the emergence of viruses with unique characteristics or with antigenic drifts or shifts [7].

As of September 2012, Indonesia had seven surveillance systems administered by Ministry of Health (MOH) that provided information about acute respiratory illness (ARI), pneumonia and influenza (Table 1). Three of these systems had hospital-based surveillance sites. Evaluations of two hospital-based sentinel surveillance systems, the sentinel pneumonia surveillance system operated by the ARI Subdirectorate since 2007 and the laboratory-based severe acute respiratory infection (SARI) surveillance system operated by the National Institute of Health Research and Development (NIHRD) since 2006, found that both had limited utility. Both systems had sites with very low surveillance activity and the sentinel pneumonia surveillance system lacked laboratory data while the SARI surveillance system lacked epidemiological focus. This led the ARI Subdirectorate and NIHRD teams to terminate these systems in December 2012 and combine efforts to establish a SARI surveillance system in 2013 that addressed their collective needs.

**Table 1:** **Indonesia’s nationally-administered surveillance systems for acute respiratory infections, pneumonia and influenza, as of September 2012**

MOH system administrator	Surveillance system	General objective	Type	Sites	Starting year	Reporting frequency	Laboratory testing
ARI subdirectorate	Sentinel pneumonia surveillance*	Monitor trends of pneumonia	Sentinel, aggregated data	40 health centers and 40 district hospital	2007	Monthly	No
ARI subdirectorate	Routine pneumonia surveillance	Program performance for pneumonia case detection	Population-based, aggregated data	All health centers	1983	Monthly	No
Surveillance subdirectorate	Early warning alert and response system	Outbreak detection	Population-based, aggregated data	All health centers	2008	Weekly	No
Surveillance subdirectorate	Monthly outbreak-prone disease surveillance	Trends for notifiable diseases	Population-based, aggregated data	All health centers and some district hospitals^#^	2003	Monthly	No
Surveillance subdirectorate	ILI syndromic surveillance	Outbreak detection	Sentinel, aggregated data	20 Health Centers	2010	Weekly	No
NIHRD	ILI virological surveillance	Influenza virus trends	Sentinel, case-based	26 Health Centers	2006	Weekly	Yes (influenza)
NIHRD	SARI virological surveillance*	Viral and bacterial trends	Sentinel, case-based	10 tertiary hospitals	2006	Monthly	Yes (viral and bacterial pathogens)

*MOH* Ministry of Health, *ILI* Influenza-like Illness; *SARI* Severe Acute Respiratory Infection, *ARI* Acute Respiratory Infection, *NIHRD* National Institutes for Health Research and Development.

*Systems were terminated in December 2012 and resources combined to create Surveilans ISPA Berat di Indonesia (SIBI).

^#^Number of district hospitals participating is unknown because hospital participation is not mandated nationally.

The new SARI system called ‘Surveilans ISPA Berat Indonesia’ (SIBI) enabled the ARI Subdirectorate to focus on the epidemiology and influenza disease control program aspects whilst NIHRD focused on the laboratory diagnostics. The objectives of SIBI were to monitor epidemiological and virological trends of influenza in different parts of Indonesia. Even though the system may detect cases of novel or emerging influenza viruses, this was not deemed the primary objective of the system as other systems such as the nationally comprehensive Early Warning Alert and Response System (EWARS) provide such function.

One aspect that had to be considered carefully in establishing SIBI was the selection of the geographic locations and hospitals to become sentinel sites. The WHO guideline highlights system stability, feasibility and representativeness as the most important factors to consider when choosing a sentinel site [6]. The guideline also suggests considering the suitability of sites to assess the disease burden of influenza whereby information about the population served by the hospital can be determined to enable disease incidence rate calculation. When multiple sentinel sites are being considered, WHO recommends selecting sites that represent different population centers or climate zones since this will provide information about transmission patterns among sub-populations with unique demographic or socio-economic characteristics. Critically, the guideline states that there is no ideal number of sentinel sites in a country but that countries should start with one or a few sentinel sites and only expand if these function well.

As Indonesia is a large archipelago nation, national decision-makers are cognizant of the regional differences in culture, religion, economic prosperity and development [8]. This results in a push for public health initiatives to have representation according to administrative divisions such as a surveillance site in each province or to apply statistical approaches for calculation of the number of sentinel sites. These approaches may not be suited or feasible for sentinel surveillance systems where the objective is to obtain high-quality patient level data to monitor regional clinical, epidemiological and microbiological disease trends. For example, having sentinel sites in contiguous provinces with similar population demographics would yield more cases enrolled into the system but would not yield additional novel information about regional variation in disease activity. Similarly, having representativeness according to population density would yield large numbers of sentinel sites due to the size of the country but would also not necessarily capture unique regional characteristics. Lastly, since influenza surveillance involves costly laboratory diagnostic testing, the larger the number of sentinel sites the more expensive the shipment and testing of specimens.

Considering the guidance from WHO and the national prerogatives, deciding on the number, location and hospitals to be selected as sentinel SIBI sites was a challenge. Previous studies have reported on lessons learnt from establishing influenza surveillance systems [9, 10], but none have reported comprehensively on the process of sentinel site selection. This study describes the process and rationale by which the six hospitals were selected as sentinel SIBI sites in Indonesia. The experience may guide other countries interested in adopting the WHO guideline and provide an approach for sentinel site selection or future surveillance system expansion.

## Methods

### Step one: creation of a hospital shortlist

**Figure 1:**
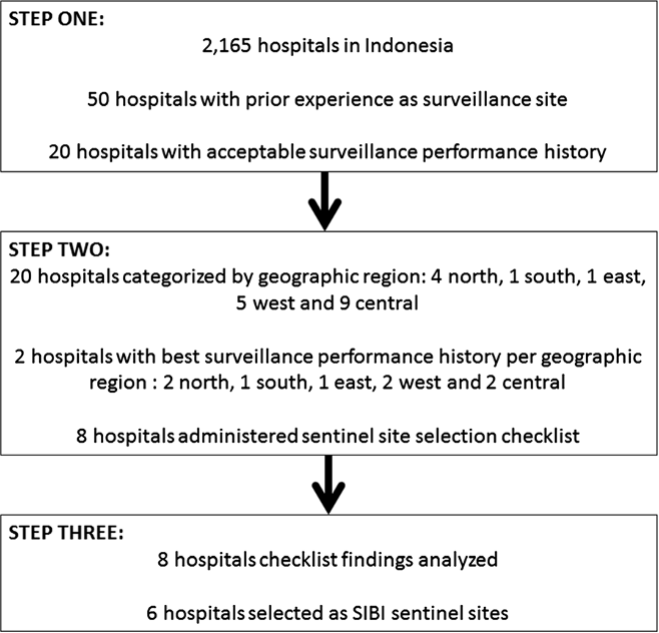
**Three-step process for SIBI sentinel site selection.**

Indonesia has 2,165 public and private hospitals [11]. Before applying the WHO guideline for sentinel site selection, we shortlisted from 2,165 hospitals to a manageable number to enable detailed assessment and final selection. To achieve this, we limited selection to public hospitals that had previously participated in the sentinel pneumonia or SARI surveillance systems. We ranked these 50 hospitals in terms of two performance indicators, timeliness and completeness of data reporting to national level. Ranking of hospitals according to their surveillance performance history enabled prioritization of good sites in subsequent steps of the selection process. Timeliness indicated the proportion of on-time reports received by MOH from the sentinel site, where 100% indicated full compliance with data reporting requirements (by the 10th of each month). Completeness indicated the number of reports made to MOH per year, where 100% indicated full compliance (12 reports received from the sentinel site per year). Timeliness and completeness data for the 50 hospitals were extracted from the MOH surveillance system databases. Hospitals with less than 50% timeliness and completeness were excluded based on poor surveillance performance history. This resulted in 20 hospitals in the shortlist from Step One (Figure 1).

### Step two: application of WHO guideline

The second step was to operationalize the WHO recommended attributes for sentinel site selection; stability, representativeness and feasibility. Since influenza disease burden has not been established in Indonesia, we incorporated this into the selection process.

For stability, the WHO guideline recommended calculating the funds needed to cover general costs of surveillance operations for the long term. We operationalized this by calculating the yearly costs of one sentinel site and determining the number of sites that could be established using funds available from MOH and donors for the next three years. Cost calculations were based on previous surveillance system experience and included one training for five hospital staff (2 doctors, 1 nurse, 1 laboratory worker and 1 medical records staff), four monitoring visits to the site, six-monthly national meetings and costs for stationery and staff incentives. Based on previous SARI surveillance experience, each sentinel site was expected to generate 150 specimens per year. Costs for specimen collection, shipment to NIHRD and testing using real-time polymerase chain reaction (RT-PCR) and virus isolation (for 10% of specimens) were also calculated.

For representativeness, we first considered geographic representation of the hospitals. We ranked the 20 hospitals from Step One according to their geographic location to represent west (Sumatera island, n = 5), north (Sulawesi island, n = 4), central (Java and Kalimantan islands, n = 9), south (East and West Nusa Tenggara islands, n = 1) and east (Maluku and Papua islands, n = 1). For hospitals in the same geographic regions, only the two top-performing hospitals were kept in the list and the others excluded. This resulted in a shortlist of eight hospitals – a number more aligned with the resources available for the surveillance system (Figure 1).

We developed a sentinel site selection checklist to address the other attributes and criteria. This included five questions to assess representativeness, such as the type of hospital (general versus specialty), accessibility of wards for influenza surveillance and the demographics of the population served by the hospital (Table 2). For feasibility, we included 13 questions to assess the motivation of the hospital to participate in the system, the infrastructure and human resource capacity available for surveillance activities (Table 2).

**Table 2:** **Checklist criteria and findings from the eight hospitals visited for sentinel site selection**

Attribute	Criteria	Maluku	North Sulawesi	East Kalimantan	South Kalimantan	North Sumatra	Yogyakarta	West Nusa Tenggara	West Kalimantan
Feasibility	1. Hospital management agreeable to influenza surveillance	Yes	Yes	Yes	No	Yes	Yes	Yes	No
	2. Two surveillance coordinating doctors available	Yes	Yes	Yes	Yes	Yes	Yes	Yes	Yes
	3. Surveillance nurse available to screen patients	Yes	Yes	Yes	Yes	Yes	Yes	Yes	Yes
	4. Surveillance laboratory staff available for specimen collection	Yes	Yes	Yes	Yes	Yes	Yes	Yes	Yes
	5. Surveillance medical records staff available to report data	Yes	Yes	Yes	Yes	Yes	Yes	Yes	Yes
	6. Refrigerator for specimen storage available	Yes	Yes	Yes	Yes	Yes	Yes	Yes	Yes
	7. Specimen courier service available	Yes	Yes	Yes	Not sure	Not sure	Not sure	Yes	Yes
	8. Computer and internet access available	Yes	Yes	Yes	Yes	Yes	Yes	Yes	Yes
	9. Frequent internet problems at hospital	Yes	Yes	No	Yes	No	No	Yes	No
	10. Adequate power supply for refrigerator	Yes	Yes	Yes	Yes	Yes	Yes	Yes	Yes
	11. Back up generator available	Yes	Yes	Yes	Yes	Yes	Yes	Yes	Yes
	12. Electronic or paper record for medical record system	Electronic	Paper	Paper	Electronic	Electronic	Electronic	Paper	Electronic
	13. ICD 10 used	Yes	Yes	Yes	Yes	Yes	Yes	Yes	Yes
Representativeness	14. District or province level hospital	Province	District	Province	District	District	District	Province	District
	15. Hospital for all age groups	Yes	Yes	Yes	Yes	Yes	Yes	Yes	Yes
	16. Hospital for all socioeconomic level populations	Yes	Yes	Yes	Yes	Yes	70% low income	60% low income	Yes
	17. All wards can participate in surveillance	Yes	Yes	Yes	Yes	Yes	Yes	Yes	Yes
	18. General or specialty hospital	General	General	General	General	General	General	General	General
Disease burden calculation	19. Hospital patient admissions per month	1100	576	1200	1000	240	920	1100	590
	20. Patient home addresses available so that hospital catchment population can be estimated	Yes	Yes	Yes	Yes	Yes	Yes	Yes	Yes
	21. X-ray conducted routinely for respiratory disease patients	Not sure	No	No	No	Yes	No	No	No

As stated by the WHO guideline, adequate patient volumes and ability to ascertain the hospital’s catchment population are needed to calculate influenza disease burden. We added three questions to the checklist to explore these issues. For patient volumes, we requested data on total patient admissions not respiratory patient admissions since hospitals may vary in their disease coding and recording systems.

Four teams comprising ARI Subdirectorate, NIHRD and Centers for Disease Control and Prevention (US-CDC) staff were trained in the sentinel site selection checklist objective and administration. Each team visited two hospitals where the teams met with the hospital director (or management representative), the medical and nursing staff and the laboratory team. These hospital personnel were targeted as they could answer the various administrative and procedural questions in the checklist. After introducing the concept of SIBI and answering any general questions, one team member administered the questionnaire and the others documented the answers. The teams then toured the hospital facilities including the medical records unit, emergency/patient admission, the laboratory and some patient wards. This enabled visual confirmation of the hospital capacity and likely flow of SARI patient enrolment into SIBI. The teams also reviewed medical records data on the number of monthly hospital admissions. Each sentinel site selection visit took 4-6 hours and all hospitals were visited in a three-week period. Funding for the sentinel site selection visits was made available by WHO.

### Step three: analysis and selection of sentinel sites

Checklist data from the eight hospitals were entered into a spreadsheet and analyzed descriptively (Table 2). The four teams met to determine which hospitals would be enrolled as SIBI sentinel sites based on the visit findings. Hospital management motivation to participate as a surveillance site was deemed a key criterion for selection as solutions to other issues could be addressed as long as the hospital was willing to collaborate. Thus, if hospital management did not welcome the surveillance activity, it no longer qualified for selection.

## Results and discussion

### Stability

The cost of establishing one sentinel site was USD 40,000 per year. Costs included training for five hospital staff (USD 5,900), specimen management and testing (USD 8,700), quarterly national monitoring missions by ARI Subdirectorate and NIHRD staff (USD 10,100), six-monthly national review meetings (USD 9,300) and administrative costs such as printing forms, hospital staff incentive payments, stationery and part-salary of a national level data manager apportioned to one-third of the person’s time (USD 5,580). From the MOH government and donor funds available and projected for the next three years, six SIBI sentinel sites could be established.

### Representativeness

Based on the geographic location of the eight hospitals visited, there was representation of east (Maluku), west (North Sumatra), north (North Sulawesi), central (Yogyakarta, Kalimantan) and south Indonesia (West Nusa Tenggara). All hospitals were general hospitals and reported coverage of all age-groups and socio-economic population groups, although the hospitals in West Nusa Tenggara and Yogyakarta reported that more than 50% of their hospital admissions were for low socioeconomic population (Table 2). All hospitals confirmed that surveillance could be conducted in all wards where SARI patients may be admitted.

### Feasibility

All eight hospitals visited had five staff available for the day-to-day operation of SIBI surveillance as well as basic infrastructure requirements such as a refrigerator, back-up power supply, computers and internet service for data reporting (Table 2). Even though four sites reported unreliable internet service at the hospital, back-up measures such as a WIFI modem could be utilized. For specimen shipment to NIHRD in Jakarta, three sites were uncertain of couriers available in the hospital vicinity. However, based on the locations of these hospitals and the NIHRD team’s experience, courier services were likely to be available. Two hospitals did not express willingness to participate in the surveillance system due to difficulties to internally coordinate surveillance activities (Table 2).

### Disease burden

All hospitals collected patient home addresses which would enable estimation of the hospital catchment population (Table 2). Six hospitals had >500 admissions per month and two reported lower admission rates (220 and 240 admissions per month for West Nusa Tenggara and North Sulawesi respectively). None of the hospitals had a standard protocol mandating chest x-rays for respiratory patients, but all hospitals estimated that 50% of patients would have x-rays requested by the treating physician. For surveillance purposes, the number of patients with x-rays generated through this approach would be large enough to enable analysis trends relating to pneumonia. Thus, for future calculation of disease burden of influenza in Indonesia, all eight hospitals were considered suitable.

## Conclusion

**Figure 2:**
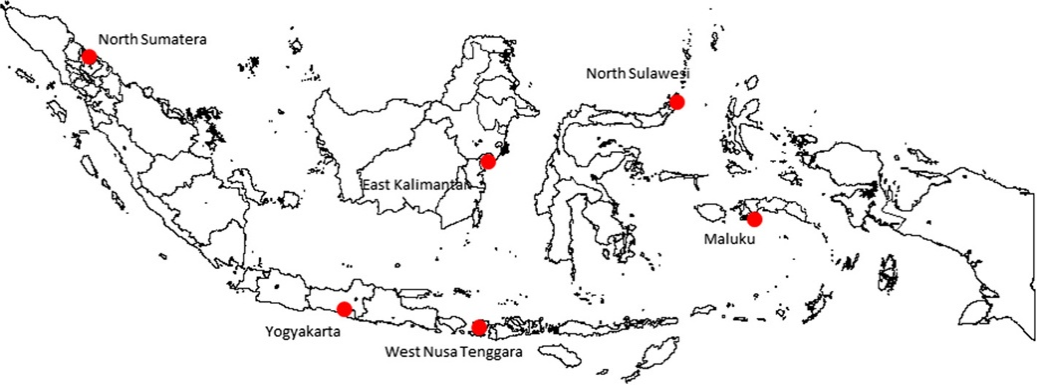
**Location of six SIBI sites established in Indonesia, 2013.**

Six hospitals were selected and enrolled as SIBI sentinel sites based on the multi-step sentinel site selection process (Figure 1). These were the hospitals in Maluku (east), North Sulawesi (north), East Kalimantan (central), North Sumatra (west), Yogyakarta (central) and West Nusa Tenggara (south, Figure 2). The lack of hospital management motivation to participate as a sentinel site was the key reason for not selecting the hospitals in South and West Kalimantan.

The WHO guideline was useful as it identified the key attributes to consider in sentinel site selection and it helped address public health decision maker concerns regarding the geographic representation of the surveillance system. However, since the WHO guideline does not provide an algorithm or scoring system for site selection, we had to operationalize the attributes into quantitative and qualitative criteria, and to prioritize these according to our context. We also had to develop our own approach for shortlisting from all the hospitals in Indonesia to a number that could be assessed using the detailed WHO approach.

Despite using a multi-step approach to sentinel site selection, there were some limitations to our process. These included reliance on self-report for checklist items such as the hospital’s motivation to participate in the surveillance system and availability of hospital staff to conduct surveillance activities. Respondents may have sought to appease the assessment team by positively biasing their answers. To address this limitation, hospitals ultimately selected as sentinel sites were asked to write a formal letter to confirm their commitment to become a surveillance site, and to list surveillance staff and their specific role in the surveillance system. For other checklist items, such as the proportion of respiratory disease patients receiving chest x-ray or the socioeconomic level of the population served by the hospital, it was not feasible to verify the answers provided by the hospital team during the assessment visit. However, these issues will be revisited in more detail once the surveillance system stabilizes and disease burden can be enumerated. Another limitation is that the sentinel site selection process does not guarantee good surveillance performance. We endeavor to maximize performance through the routine monitoring visits and twice-yearly review meetings.

As the primary objective of SARI surveillance is to monitor the epidemiological and virological characteristics of influenza, surveillance does not have to be comprehensive at all hospitals or in all provinces. A few sentinel sites may appropriately represent the situation in-country and to inform public health officials on groups that need to be targeted for preventive and treatment measures. To date, there have been no studies to investigate whether influenza patterns vary across regions in Indonesia, but since difference regions have different climatic patterns and this is known to impact influenza circulation [12, 13], it is possible that influenza patterns are different. Future studies will be required to determine whether the six SIBI sites adequately represent influenza activity in Indonesia and the extent to which the clinical, epidemiological and virological findings from the six sentinel sites are generalizable. This approach may address decision-maker concerns about surveillance representativeness in a cost-efficient manner.

As SIBI stabilizes over time and long term resources are assured, there is potential to enhance the system to also monitor other important diseases such as dengue, meningitis and severe diarrheal illness [6]. This allows for further efficiencies in utilization of sentinel surveillance systems for specific public health objectives and to minimize the burden of establishing additional stand-alone systems. The surveillance system can also be used to evaluate the benefits of public health interventions and to monitor disease severity and impact during a future influenza pandemic [14]. SIBI can also be used for emerging zoonotic diseases and allow for laboratory-based collaborations with the Ministry of Agriculture on diseases such as Nipah and Hendra virus or emerging coronavirus infections. Overall, we hope that this report clarifies the rationale for the geographic distribution, the number of sites and the methods for selection.
